# Neurological Complications following Blood Transfusions in Sickle Cell Anemia

**DOI:** 10.1155/2017/3649397

**Published:** 2017-01-03

**Authors:** Hana Alharbi, Nayaab Khawar, Jolanta Kulpa, Anne Bellin, Simona Proteasa, Revathy Sundaram

**Affiliations:** New York Methodist Hospital, Brooklyn, NY, USA

## Abstract

In Sickle Cell Anemia (SCA) patient blood transfusions are an important part of treatment for stroke and its prevention. However, blood transfusions can also lead to complications such as Reversible Posterior Leukoencephalopathy Syndrome (RPLS). This brief report highlights two cases of SCA who developed such neurological complications after a blood transfusion. RLPS should be considered as the cause of neurologic finding in patients with SCA and hypertension following a blood transfusion.

## 1. Introduction

Sickle Cell Anemia (SCA) can affect multiple organs including the brain and may lead to significant morbidity and mortality [1]. About 25% of SCA patients will have a neurologic complication [2]. Blood transfusions are an important part of the treatment of anemia. In children prophylactic red blood cell transfusions have been shown to reduce the risk of first stroke or silent stroke when transcranial Doppler (TCD) ultrasonography shows abnormal cerebral blood flow [3]. Blood transfusions reduce the risk of recurrent or silent strokes [4, 5]. Many complications of transfusion therapy are known; however neurological complications are a rare occurrence and may be unrecognized and underreported.

Blood transfusions can also cause complications such as Reversible Posterior Leukoencephalopathy Syndrome (RPLS) [6, 7]. RPLS is characterized by neuroimaging findings of vasogenic subcortical edema without infarction which are reversible. The clinical syndrome of RPLS involves headache, encephalopathy, visual symptoms, and seizures [6]. RPLS was initially described by Hinchey et al. in patients hospitalized for various acute disease entities with concomitant hypertension and renal disease. Many of the patients also received immunosuppressive therapy. Other conditions that cause RPLS include connective tissue disorders, cytotoxic drugs, malignancy, and infection [7]. Clinically RPLS produces an acute or subacute encephalopathy that occurs with a sudden increase in blood pressure (BP). Neuroimaging techniques demonstrate abnormalities involving the white matter, especially bilateral edema in the posterior portions of the cerebral hemisphere. It can also affect other areas of the cerebral cortex, spinal cord, or cerebellum [8].

We report two pediatric cases of SCA patients who developed such neurological complications. The first case developed hypertension and seizures following blood transfusions. The second case developed seizures following exchange transfusion.

## 2. Case 1

A 5-year-old male with SCA-SS variant and a history of asthma presented with fever and weakness of one-day duration and a right lower lobe pneumonia. He was transfused in the past with no complications. The complete blood count (CBC) revealed leukocytosis and hemoglobin (Hgb) of 6.8 g/dL. He developed severe chest and back pain, tachypnea, and increasing oxygen requirements. Repeated CBC revealed Hgb of 5.5 g/dL and a pretransfusion BP of 128/82 mmHg. He received 15 mL/kg of PRBC over four hours. During posttransfusion, he was noted to have an elevated blood pressure of 171/97. He received another unit of blood. The posttransfusion CBC revealed Hgb of 12.3 g/dL. The blood urea nitrogen (BUN) level was 14 mg/dL (normal 7–18 mg/dL) and creatinine value was 0.28 mg/dL (normal 0.67–1.17). Clonidine and Lasix were started.

The hypertension with the systolic pressure ranging from 160–174 and diastolic from 90–101 was persistent. He complained of headache and vomited once. He had a staring episode with stiffening extremities and was unresponsive to painful stimuli.

CT of the brain was normal. Prior TCD was normal. BP remained elevated and he had a second seizure. An exchange transfusion was performed. Over the following days his BP normalized and his overall condition improved with no further seizures. MRI/MRA of the brain and neck was obtained and revealed stenosis of the right middle cerebral artery and left posterior cerebral artery, old ischemic changes, and chronic lacunar infarct with no new infarcts. He was started on a chronic transfusion program. Six months later he was stable with no further seizures.

## 3. Case 2

An eight-year-old female with SCA-SS type was admitted with fever, headache, nausea, and abdominal pain. Her BP was of 109/49 mmHg. She was transfused on past occasions. Her baseline Hgb was 7.9 g/dL. The BUN level was 8 mg/dL (normal 7–18 mg/dL) and creatinine was 0.40 mg/dL (normal 0.67–1.17). She developed elevated bilirubin of 24.6 mg/dL suggestive of intrahepatic cholestasis. Her Hgb dropped to 6.8 g/dL and she received a transfusion of PRBC. Her headache persisted and MRI of the brain was normal. As the hyperbilirubinemia increased and her Hgb dropped further she underwent an exchange transfusion. She tolerated this well. Ten hours later she became unresponsive with elevation of the BP to 150/100 mm/Hg.

**Figure 1:**
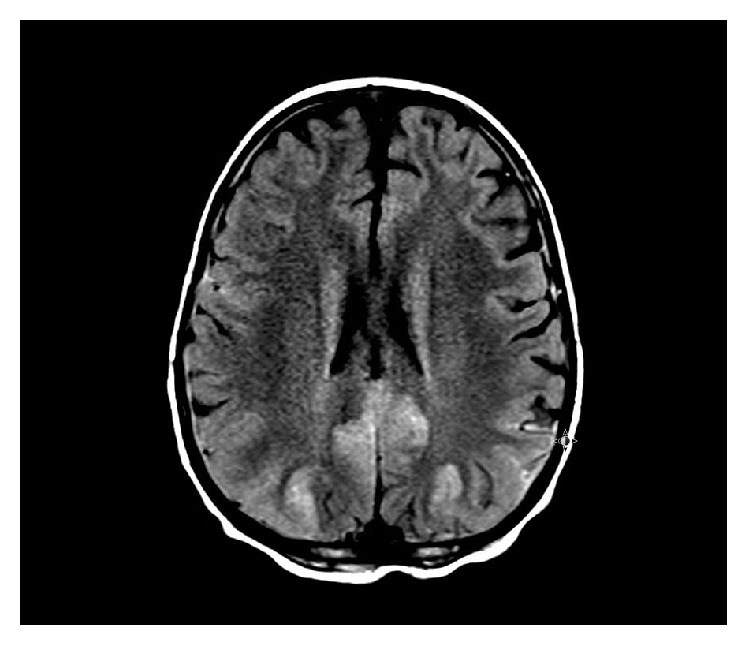
Brain MRI: T2 hyperintensity involving the cortex and possibly subcortical white matter at the occipital and posterior parietal lobes, bilaterally and fairly symmetrically.

CT of the brain revealed a small epidural hematoma with no evidence of mass effect or midline shift. An MRI revealed symmetrical T2 signal hyperintensity in the occipital and posterior lobes consistent with RPLS (Figure 1). The patient was started on Labetalol for hypertension. Her clinical condition improved over the next few days and she was discharged home with no neurological deficit.

## 4. Discussion

RPLS is a rare complication following blood transfusion with only a few cases reported in the literature [9]. Common etiologies include hypertension, eclampsia, and calcineurin inhibitor use [6]. Comorbidities include hypertension, renal disease, dialysis, and transplantation [6]. The most common signs and symptoms include headache, vomiting, confusion, and altered level of consciousness ranging from drowsiness to stupor. There can be diminished spontaneity of speech and abnormalities of visual perception [9].

RPLS was first described in 15 patients who developed clinical features and had confirmatory radiological findings [10].The prevalence of RPLS in the pediatric population is not well established and has only been reported in children following chemotherapy and tumor lysis syndrome and in children with hypertension [11].

We present two pediatric patients with SCA who developed elevated BP and neurological events following blood transfusions. There was no history of preexisting hypertension. RPLS was confirmed radiologically in one patient and suggestive in the other. A review of the literature reveals that symptoms develop when the Hgb levels increased by at least 5 g/dL following blood transfusion [7, 9, 12, 13]. The second patient was transfused for a low Hgb. Following the transfusion she developed neurological signs and imaging studies confirmed RPLS. She was managed with supportive care and BP control. The lower than expected rise in Hgb following 2 units could be explained by ongoing hemolysis related to sickling as evidence by a reticulocyte event of 14% hemodilution secondary to fluid overload which may also have had a part.

Preexisting hypertension associated with RPLS was not reported in the literature. One previous case reported an acute elevation of BP after blood transfusion [12]. Multiple transfusions can become complicated with increase in BP and coagulopathy [14]. Posttransfusion hypertension, convulsion, and cerebral hemorrhage were reported in patients with thalassemia major [15]. This was related to increase in blood viscosity which correlated with the increase in hematocrit level. Blood viscosity plays a role in the pathophysiology of Sickle Cell Anemia. In both cases the BP was significantly elevated after transfusion followed by neurological events. A common clinical hypertensive encephalopathy is the cause of RPLS [10]. Sudden elevation in systemic BP occurs and exceeds the autoregulatory capability of the cerebral vasculature and results in breakdown of the blood brain barrier or possibly a brain-capillary leak. The cause of brain-capillary leak syndrome may be due to hypertension, fluid retention, and endothelial dysfunction [10]. The increased volume of intravenous fluid usually administered to patients with vasoocclusive crisis may be a contributing factor.

Blood transfusions are administered in a prophylactic manner in selected patients for stroke prevention. In the acute setting transfusions are used frequently to treat acute chest syndrome and severe anemia. While lifesaving, blood transfusions may also lead to serious complications. The inherent nature of Sickle Cell Anemia, in particular vascular endothelial damage and the propensity to hypercoagulopathy, contributes to the development of these complications. Neurological symptoms associated with elevated BP and transfusions should raise suspicion for the clinical entity of RPLS.

Blood transfusions should be used judiciously with attention paid to the volume and rate of administration. A rapid rise in the hemoglobin level should be avoided as this can increase blood viscosity and lead to neurological complications such as RPLS. We also recommend early exchange transfusion in cases where there is a rise in Hgb values more than 5 gm/dL from pretransfusion levels to reduce viscosity and thus minimize the risk of neurological complications. BP should also be closely monitored as this may be an early and important indicator of RPLS.
